# Innovative nomogram for predictive risk stratification of aspiration pneumonia in post-stroke dysphagia patients

**DOI:** 10.3389/fneur.2025.1556541

**Published:** 2025-06-03

**Authors:** Junming Wang, Pengfei Wang, Zhengyao Shen, Kehan Liao, Daikun He, Zhigang Pan

**Affiliations:** ^1^Department of General Practice, Jinshan Hospital, Fudan University, Shanghai, China; ^2^Center of Emergency and Critical Care Medicine, Jinshan Hospital, Fudan University, Shanghai, China; ^3^Research Center for Chemical Injury, Emergency and Critical Medicine of Fudan University, Shanghai, China; ^4^Key Laboratory of Chemical Injury, Emergency and Critical Medicine of Shanghai Municipal Health Commission, Shanghai, China; ^5^Department of General Practice, Zhongshan Hospital, Fudan University, Shanghai, China

**Keywords:** post-stroke dysphagia, aspiration pneumonia, risk prediction, nomogram model, stroke outcomes

## Abstract

**Background:**

Post-stroke dysphagia (PSD) affects up to 76% of stroke patients and increases aspiration pneumonia (AP) risk, leading to higher mortality among older survivors. Current risk assessment tools for AP in PSD patients lack precision.

**Methods:**

We conducted a retrospective study of 7,134 stroke patients admitted to Jinshan Hospital from 2019 to 2023. We used multivariable logistic regression to identify AP predictors and constructed a nomogram model using these predictors. Model performance was evaluated using bootstrap resampling, calibration, and decision curve analysis. Internal validation was conducted on 30% of cases, and external validation was performed on 500 PSD patients from community health centers.

**Results:**

Among 2,663 PSD patients, 578 (21.7%) developed AP. Independent predictors included age, stroke severity, hyperlipidemia, hyperhomocysteinemia, heart failure, CRP, WBC, neutrophil ratio, Hb, FBG, prealbumin, BNP, and serum sodium. The nomogram model showed excellent discrimination (C-index: 0.885) and good agreement between predicted and observed AP probabilities. It provided net benefit across various threshold probabilities.

**Conclusion:**

Our study developed the first dedicated nomogram for AP risk prediction in PSD patients, incorporating novel predictor combinations and demonstrating robust validation across multi-center cohorts. This fills an important clinical need under community conditions by enabling early identification of high-risk PSD patients using routinely available clinical variables.

## 1 Introduction

Post-stroke brain function often exhibits varying degrees of impairment, encompassing physical disabilities, language disorders, altered consciousness, dysphagia, and others. Among these, Post-Stroke Dysphagia (PSD) is a frequently overlooked yet significant manifestation of brain dysfunction following acute stroke, severely impacting patients' rehabilitation and quality of life (1). Recent data suggest that PSD prevalence ranges from 40 to 81% (2, 3), with four-fifths of PSD patients still exhibiting dysphagia at discharge (4). Literature reports indicate that 15 to 41% of acute stroke patients suffer from persistent dysphagia (5).

PSD patients often experience Aspiration Pneumonia (AP) during feeding (6). AP complications prolong hospital stays, intensive care unit (ICU) admissions, and mortality, imposing a heavy burden (7, 8). Large-scale clinical trials on AP prevention are crucial (9). While meta-analyses suggest swallowing therapy may help, more high-quality interventions are needed (10). Dysphagia, a predictor of poor prognosis and an AP risk factor, can persist years post-stroke (5, 11, 12). PSD patients have a high AP risk due to potential neural damage affecting swallowing (13, 14). A cohort study estimates 18.75% PSD patients develop AP, influenced by factors like age, stroke severity, and comorbidities (15, 16). Hence, a predictive model considering clinical and laboratory indicators is urgently needed for early AP prevention and treatment in PSD patients.

In assessing PSD-related AP during acute stroke using standardized, effective, and reliable tools, factors such as gender, stroke type, prior stroke, severe stroke, and diabetes should be considered to aid in disease prevention and management, thereby reducing mortality (3). To date, there have been no reports on the use of nomogram tools to construct a risk prediction model for AP in PSD patients.

Nomograms, prediction models in medicine, use logistic regression on clinical data to integrate multiple variables into a chart, predicting disease incidence, mortality, treatment effects, etc. (17, 18), with advantages over traditional models in understandability, visualization, and precision (19). By considering age, consciousness, dysphagia, smoking, stroke history, cough strength, and comorbidities like diabetes and hypertension, a nomogram can assess AP risk in stroke patients (20, 21). Clinicians use this model to calculate a composite score, estimating the probability of AP.

This study aims to develop and validate a nomogram-based risk prediction model for AP in PSD patients, identifying high-risks early to reduce AP incidence, mortality, and enhance quality of life. The model offers personalized recommendations for clinicians and promotes self-management, serving as a vital reference for PSD patient care transitions from hospitals to communitie.

## 2 Material and methods

### 2.1 Patients

**Figure 1:**
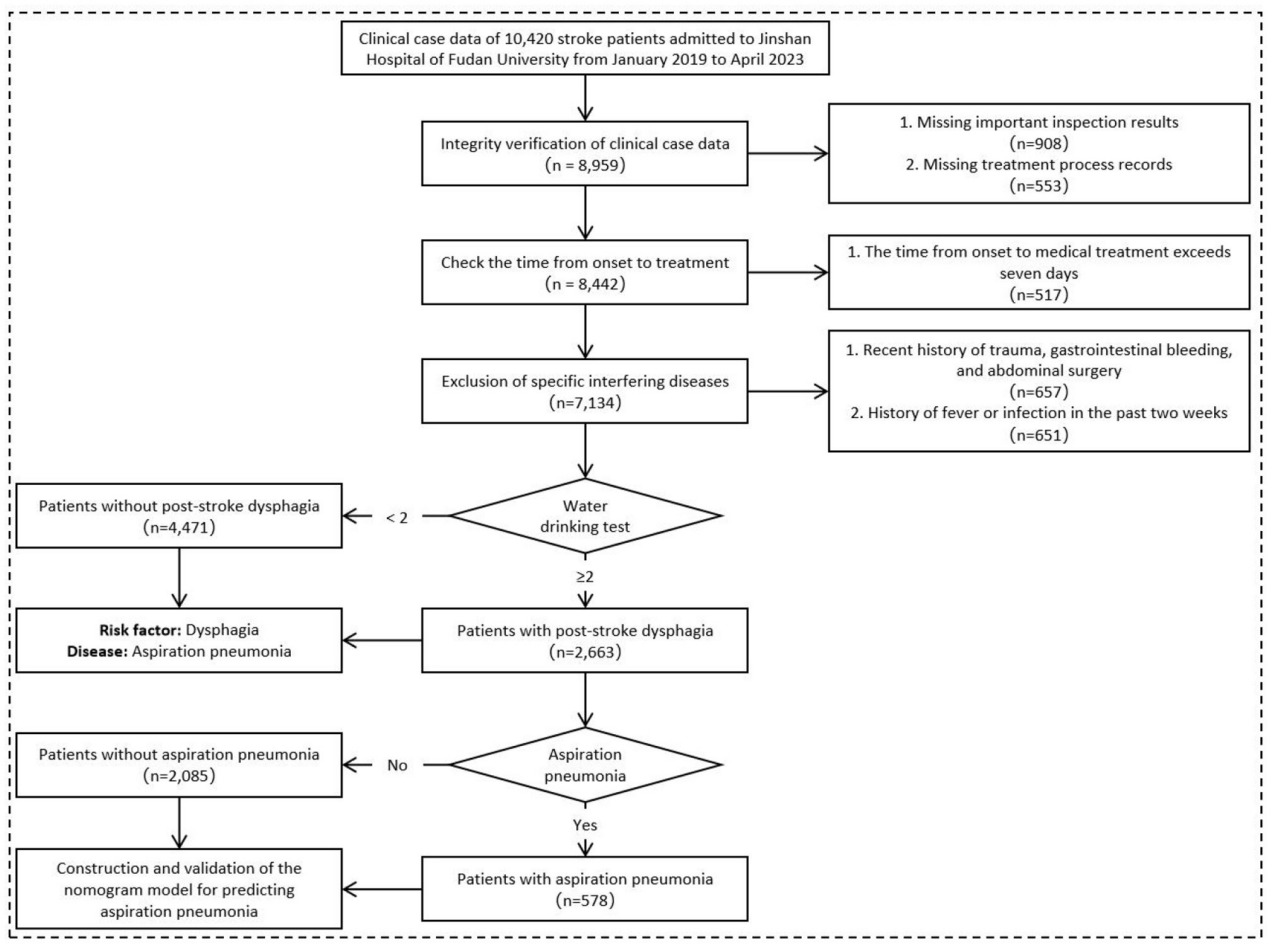
Flow chart for collecting patients' clinical case data.

A retrospective collection of 7,134 clinical case records of stroke patients, including 4,298 males and 2,836 females, was conducted. Dysphagia was identified as the most significant risk factor for AP in stroke patients. Subsequently, a subset of 2,663 acute stroke patients with clinically confirmed dysphagia (Water Swallowing Test score ≥2, administered by trained nurses using a standardized 30 mL protocol) was used to construct a Nomogram model. These selected subjects comprised a research cohort of consecutive PSD patients hospitalized in Jinshan hospital of Fudan University from January 2019 to April 2023. The entire cohort was randomly divided into a training cohort (*n* = 1,864) and an internal validation cohort (*n* = 799) at a ratio of 7:3. Based on the occurrence of AP, the patients were further categorized into a Non-AP group and an AP group (Figure 1).

### 2.2 Inclusion and exclusion criteria

#### 2.2.1 Inclusion criteria

(1) Aged 18 or above; meeting the diagnostic criteria for acute stroke according to the “Guidelines for the Diagnosis and Treatment of Acute Ischemic Stroke in China 2018” (22) and the “Guidelines for the Diagnosis and Treatment of Cerebral Hemorrhage in China (2019)” (23), with clinical manifestations of brain dysfunction, and confirmed as stroke (including hemorrhagic and ischemic) by cranial computed tomography (CT) or magnetic resonance imaging (MRI) imaging examination, and the first onset;
(2) Through the first water drinking test grading upon admission, the swallowing function is assessed as grade 2 to 5, indicating suspected or existing swallowing disorders;
(3) Complete data including at least: clinical characters such as AP, age, type of stroke, national institute of health stroke scale (NIHSS) score, encephalatrophy, diabetes, hyperlipidemia, hyperuricemia, hyperhomocysteinemia, coronary heart disease, atrial fibrillation, cardiac insufficiency, pulmonary underlying diseases, hepatic insufficiency and renal insufficiency; Laboratory indicators such as c-reactive protein (CRP), white blood cell (WBC), neutrophil% (NE%), lymphocyte% (LY%), NE, LY, red blood cell (RBC), hemoglobin (Hb), hematocrit (Hct), platelet (Plt), fasting blood glucose (FBG), total cholesterol (TC), triglyceride (TG), low-density lipoprotein (LDL), alanine aminotransferase (ALT), total protein (TP), albumin (Alb), prealbumin (PA), serum creatinine (Scr), glomerular filtration rate (GFR), brain natriuretic peptide (BNP), K^+^ and Na^+^.

#### 2.2.2 Exclusion criteria

(1) Presence of infection (including respiratory, urinary, digestive systems, etc.) before admission or within 48 h of admission, or having been diagnosed with AP, with a history of fever or infection within the past 2 weeks. Rationale: to exclude pre-existing infections that could confound AP diagnosis and ensure observed AP represents new-onset events post-stroke;
(2) Time from onset to consultation exceeding 7 days. Rationale: focuses on acute-phase dysphagia assessment, as AP risk is highest in the first week post-stroke;
(3) Recent history of significant head trauma, gastrointestinal bleeding, or abdominal surgery. Rationale: eliminates alternative causes of aspiration (e.g., traumatic dysphagia, surgical complications) unrelated to stroke pathophysiology;
(4) Other exclusion criteria, such as incomplete baseline data or missing follow-up records, that made the patient unsuitable for inclusion in this retrospective analysis. Rationale: ensures data completeness for reliable retrospective analysis of AP risk factors and outcomes.

### 2.3 Diagnostic criteria

(1) Acute stroke diagnosis: the diagnostic criteria for acute ischemic stroke require: ① Acute onset (<72 h); ② Focal neurological deficits (or, in rare cases, global neurological impairment); ③ Symptoms and signs persisting for several hours (patients undergoing thrombolysis should meet the relevant eligibility criteria); ④ Absence of intracranial hemorrhage or other pathologies on brain CT or MRI; ⑤ Presence of a causative infarct lesion on brain CT or MRI; The diagnostic criteria for acute hemorrhagic stroke require: ① Acute onset (< 72 h); ② Focal neurological deficits (or, less commonly, global neurological impairment), often accompanied by headache, vomiting, elevated blood pressure, and varying degrees of consciousness disturbance; ③ Presence of hemorrhage on brain CT or MRI; ④ Exclusion of non-vascular intracranial pathologies.
(2) AP diagnosis: ① Imaging confirmation: new pulmonary infiltrate(s) on chest X-ray or CT, typically in dependent lung zones (e.g., posterior segments of upper lobes or superior segments of lower lobes); ② Clinical criteria (≥2 of the following): fever (>38°C) or hypothermia (< 36°C); Leukocytosis (>11 × 10^9^/L) or leukopenia (< 4 × 10^9^/L); Purulent tracheal/bronchial secretions; Hypoxemia (SpO_2_ < 90% on room air or PaO_2_ < 60 mmHg); ③ Supportive evidence: documented aspiration risk (e.g., dysphagia on videofluroscopic swallowing study (VFSS) / fiberoptic endoscopic evaluation of swallowing (FEES), impaired consciousness, or witnessed aspiration event); ④ Exclusion of alternative diagnoses (e.g., pulmonary embolism, chemical pneumonitis, or non-infectious infiltrates).
(3) **Dysphagia diagnosis:** the water drinking test was performed within 24 h of admission, with patients seated upright. Instruct the patient to drink 30 mL of room temperature water at once and observe the patient's swallowing condition. The test was conducted by at least 2 well-trained nurses. If inconsistent observation results occurred, the test should be repeated. The water drinking test grades swallowing disorders: ① Grade 1: no coughing, swallowed in 5 s; ② Grade 2: no coughing, 5–10 s, needs 2 gulps; ③ Grade 3: coughing, 5–10 s, 1 gulp; ④ Grade 4: coughing, 5–10 s, >2 gulps; ⑤ Grade 5: unable to swallow in 10 s, multiple coughing. The 5-grade scale reflects increasing aspiration risk (Grade 1 = normal; Grade 5 = severe dysfunction), with Grades ≥3 indicating clinically significant swallowing disorders requiring intervention.
(4) **Encephalatrophy diagnosis:** ① Head MRI shows atrophy of brain tissue and enlargement of ventricles; ② The intracranial volume reduction (ICV)/brain volume reduction (BV) index decreases, meaning that the patient's brain volume is greater or < 2 standard deviations from normal compared to age and gender; ③ Patients with brain atrophy who meet the above diagnostic criteria have no other neurological disorders, severe organic heart and lung diseases, mental illnesses, or a history of head trauma.
(5) **Hypertension diagnosis:** in the absence of antihypertensive drugs, there were three times when the blood pressure values in the clinic were higher than normal, that is, the systolic blood pressure (commonly known as high pressure) in the clinic was ≥140 mmHg and/or the diastolic blood pressure (commonly known as low pressure) was ≥90 mmHg, and these three blood pressure measurements were not taken on the same day.
(6) **Diabetes diagnosis:** ① Fasting blood glucose ≥7.0 mmol/L (126 mg/dL); ② Random blood glucose ≥11.1 mmol/L (200 mg/dL), accompanied by typical diabetes symptoms (such as polydipsia, polyuria, weight loss, etc.); ③ Oral 75 g glucose tolerance test (OGTT) with 2-h blood glucose levels ≥11.1 mmol/L (200 mg/dL).
(7) **Hyperlipidemia diagnosis:** ① TC level ≥ 6.22 mmol/L (240 mg/dL); ② LDL-C level ≥ 4.14 mmol/L (160 mg/dL); ③ TG levels ≥ 2.26 mmol/L (200 mg/dL).
(8) **Hyperuricemia diagnosis:** when the serum uric acid level exceeds 420 μmol/L (~7 mg/dl) twice on the same day, it is considered hyperuricemia.
(9) **Hyperhomocysteinemia diagnosis:** hyperhomocysteinemia is defined as total homocysteine in the blood ≥10 μmol/L, where: ① 10–15 μmol/L is considered mild hyperhomocysteinemia; ② 15–30 μmol/L is moderate hyperhomocysteinemia; ③ A concentration >30 μmol/L is considered severe hyperhomocysteinemia.
(10) **Coronary heart disease diagnosis:** ① Having typical symptoms of angina pectoris; ② In electrocardiogram examination, changes such as ST segment descent, T wave inversion, or Q wave appearance are observed; ③ Myocardial enzyme examination shows myocardial injury; ④ Imaging examination shows the presence of coronary artery stenosis or myocardial perfusion abnormalities.
(11) **Renal insufficiency diagnosis:** chronic renal insufficiency is defined as an estimated GFR > 90 ml/min.
(12) **Hepatic insufficiency diagnosis:** ① Symptoms of liver dysfunction such as jaundice, skin itching, nausea, vomiting, and fatigue; ② Elevated levels of serum bilirubin, transaminase like ALT, aspartate transaminase (AST), alkaline phosphatase (ALP), and decreased levels of albumin and prothrombin time (PT); ③ Imaging examinations: B-ultrasound, CT, MRI and other imaging examinations reveal significant abnormalities or liver dysfunction.

### 2.4 Data extraction

The hospital's EHR system was queried for diagnoses (ICD-9 pre-Oct 2020, ICD-10 post) and exported to Excel. Data underwent screening, cleaning, and organization by 4 interns, cross-checked by 2 physicians, and finally audited by the Medical Statistics Department. Baseline characteristics and risk factors were analyzed. Blood samples were collected within 24 h: Tertiary Hospital used Aristo (Shenzhen Guosai), Sysmex (Japan), and AU5800 (Beckman) for CRP, blood routine, and biochemistry; Community Health Center sent samples to Jinshan Tinglin Hospital for analysis.

### 2.5 Statistical methods

We utilized STATA 18.0 for initial data analysis and R 4.2.3 for nomogram development and validation, leveraging the strengths of each software platform. In univariate analysis, categorical data were analyzed by Chi-square tests, while continuous data were analyzed by *t*-tests or Kruskal-Wallis tests depending on their distribution. Missing data were not imputed and statistical significance was set at *P* < 0.05. Then Logistic regression was used to identify factors affecting AP in PSD patients. A nomogram model was developed using R and the “rms” package, validated using Bootstrap, and assessed for accuracy, stability, and clinical performance through decision curve analysis (DCA) and receiver operating characteristic (ROC) analysis.

## 3 Results

### 3.1 Dysphagia is an independent risk factor for AP in stroke patients

Among the 7,134 enrolled patients with acute stroke, 804 cases met the diagnostic criteria for aspiration pneumonia (case group), while 6,330 served as controls. Comparative analysis of baseline clinical characteristics between the aspiration pneumonia group and the control group revealed statistically significant differences (*P* < 0.05) in the distribution of the following features: age, stroke type, Watian water swallow test score, NIHSS score, cerebral atrophy, hyperlipidemia, hyperhomocysteinemia, coronary heart disease, atrial fibrillation, cardiac insufficiency, pre-existing pulmonary disease, hepatic insufficiency, and renal insufficiency (Supplementary Table 1).

**Figure 2:**
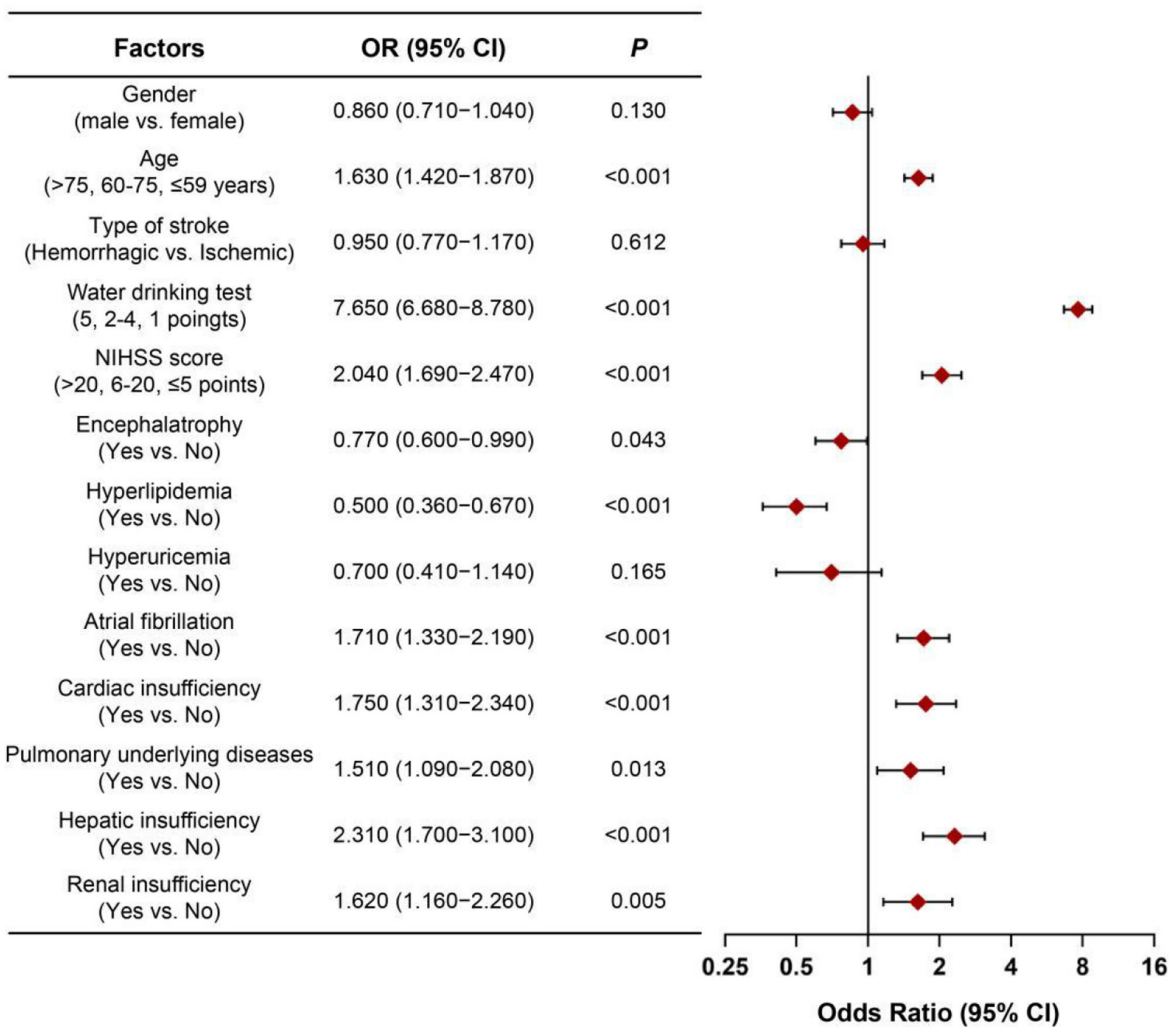
Forest plot of OR values for various influencing factors of aspiration pneumonia in stroke patients. OR, odds ratio; NIHSS score, national institute of health stroke scale score.

Clinical variables were assigned values (Supplementary Table 2). multivariable Logistic regression was used to identify factors affecting AP in stroke patients. Variables were selected by stepwise regression with Akaike information criterion (AIC) optimization. Key independent factors included age, swallowing impairment, NIHSS score, brain atrophy, hyperlipidemia, atrial fibrillation, cardiac/lung/liver/renal insufficiency. Dysphagia [odds ratio (OR) = 7.65, 95% CI: 6.68–8.78, *P* < 0.001] was the most significant risk factor (Figure 2). The AIC value was −3,258.12.

### 3.2 Clinical baseline features of PSD patients

Due to dysphagia being the most significant independent risk factor for AP in stroke patients, we then focused on a subset of 2,663 PSD patients in subsequent analysis. Compared with the Non-AP group, the AP group had statistically significant differences in the composition ratios of age, stroke type, length of hospital stay, way of discharge, NIHSS score, encephalatrophy, diabetes, hyperlipidemia, hyperuricemia, hyperhomocysteinemia, coronary heart disease, atrial fibrillation, cardiac, hepatic and renal insufficiency (*P* < 0.05; Supplementary Table 3).

### 3.3 Comparison of laboratory examinations indicators between two groups of PSD patients

Compared with the Non-AP group, the levels of CRP, WBC, NE%, NE, NE/LY, WBC/RBC, FBG, ALT, Scr, BNP, K^+^ and Na^+^ in the AP group were significantly elevated, with statistically significant differences (*P* < 0.05; Supplementary Table 4).

### 3.4 Analysis of factors influencing AP in PSD patients

With AP as the dependent variable (assignment: Non-AP = 0, AP = 1), influential factors with significant differences in baseline data were selected as independent variables to establish a Logistic regression model.

**Figure 3:**
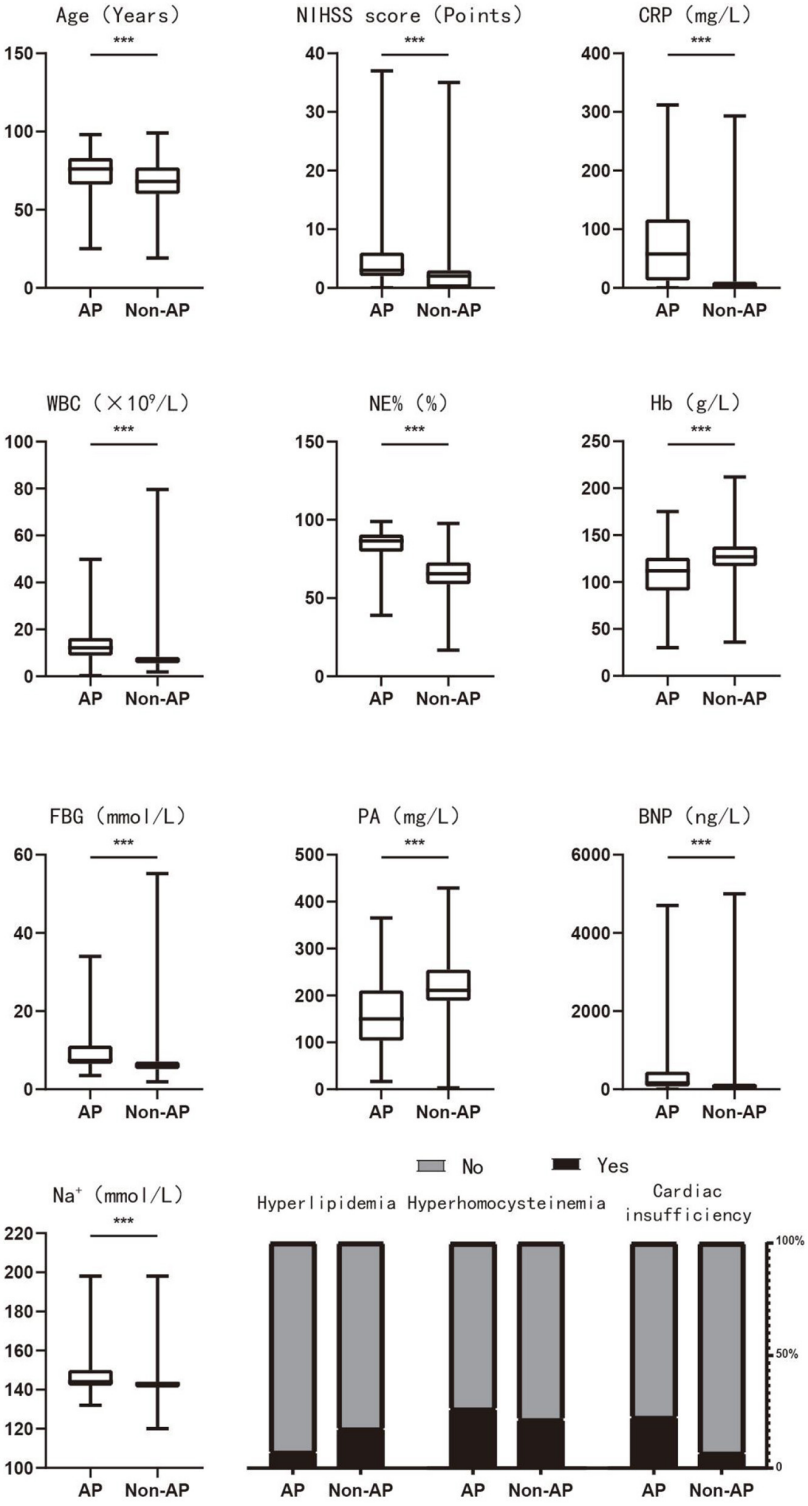
The distribution of 13 independent influencing factors in the AP and Non-AP groups of the overall cohort. AP, aspiration pneumonia; NIHSS score, national institute of health stroke scale score; CRP, c-reactive protein; WBC, white blood cells; NE, neutrophil; Hb, hemoglobin; FBG, fasting blood glucose; PA, prealbumin; BNP, brain natriuretic peptide. *P < 0.05; **P < 0.01; ***P < 0.001.

The inclusion criterion for independent variables was *P* < 0.05. A stepwise selection analysis (combining forward selection with backward elimination) was performed on the variables, and the results showed that age, NIHSS score, hyperlipidemia, hyperhomocysteinemia, cardiac insufficiency, CRP, WBC, NE%, Hb, FBG, PA, BNP, and Na^+^ were independent influencing factors for the occurrence of AP in PSD patients (*P* < 0.05), as shown in Table 1. Figure 3 demonstrates the distribution of these 13 independent influencing factors in the AP group and the Non-AP group within the overall cohort.

**Table 1:** Multivariable logistic regression analysis of AP occurrence in PSD patients.

**No**.	**Factors**	**β**	**Wald**	***P***	**AOR (95% CI)**
**Clinical characters**					
1	Age	0.222	6.996	0.008	1.249 (1.060–1.474)
2	NIHSS score	0.741	40.431	< 0.001	2.099 (1.670–2.640)
3	Hyperlipidemia (Yes vs. No)	−1.065	24.840	< 0.001	0.345 (0.223–0.516)
4	Hyperhomocysteinemia (Yes vs. No)	0.775	32.818	< 0.001	2.171 (1.663–2.827)
5	Cardiac insufficiency (Yes vs. No)	1.167	46.165	< 0.001	3.212 (2.292–4.497)
**Laboratory indicators**					
1	CRP	0.222	38.439	< 0.001	1.249 (1.164–1.340)
2	WBC	0.726	16.543	< 0.001	2.066 (1.456–2.932)
3	NE%	0.882	20.746	< 0.001	2.415 (1.653–3.533)
4	Hb	0.249	6.527	0.011	1.283 (1.060–1.554)
5	FBG	0.452	8.267	0.004	1.571 (1.155–2.140)
6	PA	0.282	17.634	< 0.001	1.326 (1.162–1.513)
7	BNP	0.340	12.214	< 0.001	1.405 (1.161–1.700)
8	Na^+^	0.350	4.689	0.030	1.420 (1.032–1.946)

AOR, adjusted odds ratio; NIHSS score, national institute of health stroke scale score; CRP, c-reactive protein; WBC, white blood cells; NE, neutrophil; Hb, hemoglobin; FBG, fasting blood glucose; PA, prealbumin; BNP, brain natriuretic peptide.

### 3.5 Construction of nomogram model

**Figure 4:**
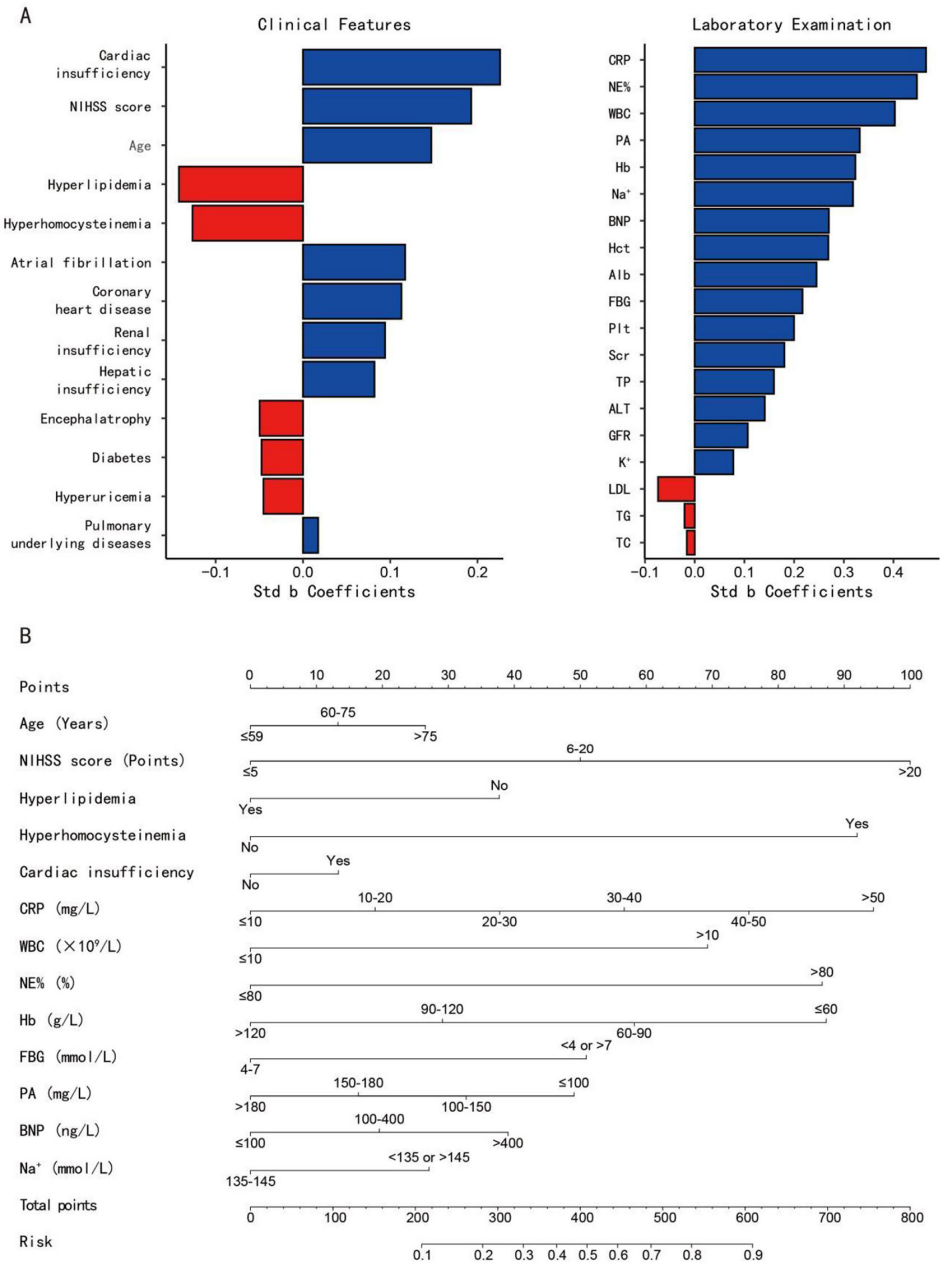
**(A)** The tornado charts display the sensitivity analysis of all variables, ranked by the absolute values of their standardized beta (std b) coefficients (horizontal axis). Variables are ordered vertically by their contribution magnitude (|std b coefficients|), with longer bars indicating stronger associations with the outcome (aspiration pneumonia); **(B)** A nomogram prediction model for the risk of AP in PSD patients. *Example: to predict the occurance risk of aspiration pneumonia for a male stroke patient of 40 with only hyperhomocysteinemia history. His NIHSS score is 3 and his laboratory test results are as follow: CRP: 69.49 mg/L, WBC: 12.2* × *10*^9^*/L, NE%: 49.9%, Hb: 133 g/L, FBG: 6.52 mmol/L, PA: 311 mg/L, BNP: 145.78 ng/L, Na*^+^*: 144 mmol/L. (1) Locate each variable's value on the corresponding axis and draw a vertical line to the 'Points' axis (Age: 0, NIHSS score: 0, Hyperlipidemia: 37.5, Hyperhomocysteinemia: 90, Cardiac insufficiency: 0, CRP: 95, WBC: 70, NE%: 0, Hb: 0, FBG: 0, PA: 0, BNP: 20, Na*^+^*: 0); (2) Sum all points (37.5* + *90* + *95* + *70* + *20* = *312.5); (3) Locate the total points on the “Total Points” axis and draw a vertical line to the bottom axis to read the predicted probability (312.5 total points* ≈ *27% risk)*. std b, standardized beta; NIHSS score, national institute of health stroke scale score; CRP, c-reactive protein; NE, neutrophil; WBC, white blood cells; PA, prealbumin; Hb, hemoglobin; BNP, brain natriuretic peptide; Hct, hematocrit; Alb, albumin; FBG, fasting blood glucose; Plt, platelet; Scr, serum creatinine; TP, total protein; ALT, alanine aminotransferase; GFR, glomerular filtration rate; LDL, low-density lipoprotein; TG, triglyceride; TC, total cholesterol.

A tornado chart revealed that these above-mentioned 13 variables are highly correlated with AP occurrence in both clinical and lab exams (Figure 4A). The variables selected for nomogram construction ranked for: (A left) Clinical indicators: top 5 sensitive clinical predictors. (A right) Laboratory indicators: top 7 and the tenth sensitive laboratory predictors. A nomogram model was built based on these indicators (Figure 4B), listing scores for each risk factor. The regression equation for AP prediction probability is as follow: P(AP) = −4.056 + 0.145 ^*^ age + 0.547 ^*^ NIHSS score −0.413 ^*^ hyperlipidemia + 1.007 ^*^ hyperhomocysteinemia + 0.146 ^*^ cardiac insufficiency + 0.207 ^*^ CRP + 0.759 ^*^ WBC + 0.949 ^*^ NE% + 0.318 ^*^ Hb + 0.557 ^*^ FBG + 0.179 ^*^ PA + 0.214 ^*^ BNP + 0.296 ^*^ Na^+^. By calculating specific scores for each independent risk factor, a total risk score is ultimately derived. Based on the correspondence between the total score and the predicted value, the probability of AP occurrence in PSD patients can be accurately predicted.

### 3.6 Internal validation of nomogram model

**Figure 5:**
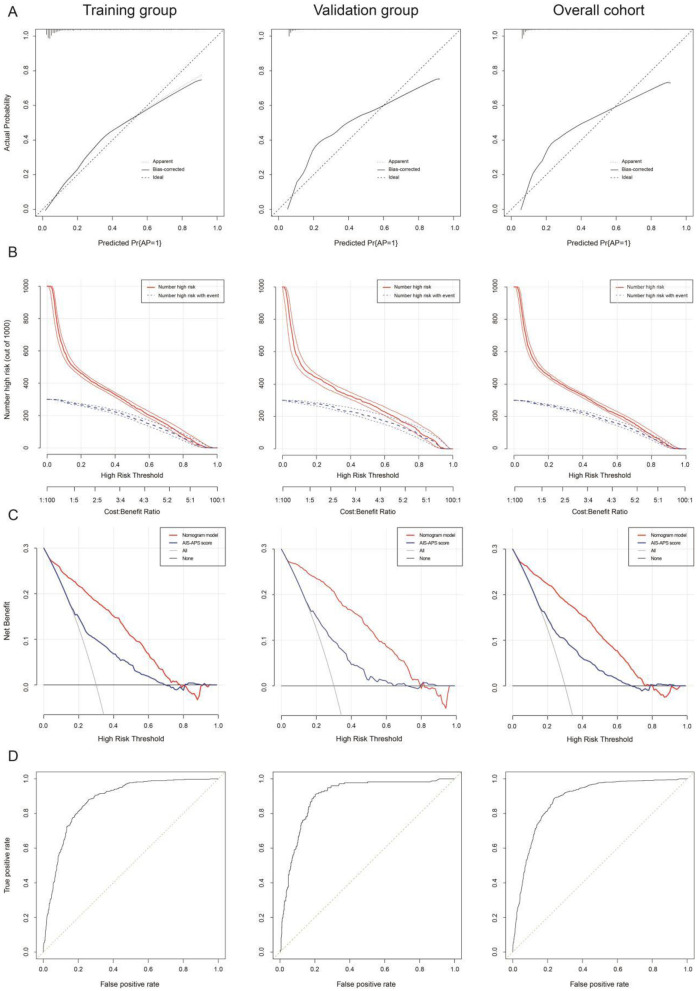
The calibration curve **(A)**, clinical impact curve (“Number high risk” = total number of patients identified as high-risk by nomogram model; “Number high risk with event” = subset of high-risk patients who actually developed AP) **(B)**, clinical decision curve [Models compared: nomogram model (age, NIHSS score, hyperlipidemia, hyperhomocysteinemia, cardiac insufficiency, CRP, WBC, NE%, Hb, FBG, PA, BNP, and Na^+^), AIS-APS score (age, atrial fibrillation, cardiac insufficiency, COPD, smoke, stoke history, Glasgow score, dysphagia, stroke type and FGB), and All model (assuming all patients receive treatment).] **(C)**, and ROC curve **(D)** in the training queue, internal validation queue, and overall cohort of nomogram. AP, aspiration pneumonia; AIS-APS score, acute ischemic stroke-associated pneumonia score.

No statistically significant difference was observed between the training and validation cohorts (*P* > 0.05), as shown in Supplementary Table 5. After 1,000 resamplings of the original data using the Bootstrap method for internal validation, the study revealed consistency indices of 0.885 (95% CI: 0.862–0.908), 0.878 (95% CI: 0.864–0.893), and 0.879 (95% CI: 0.864–0.894) for the training, validation, and overall cohorts, respectively, indicating good stability and consistency across cohorts. The calibration curve analysis showed minimal error between actual and predicted AP risks in PSD patients (Figure 5A). The clinical impact curve indicated a strong match between predicted high-risk and actual AP cases when the threshold probability exceeded 20% (Figure 5B). Clinical decision analysis revealed that the nomogram model provided more net clinical benefit than AIS-APS score when threshold probabilities ranged from 8 to 80%, 9 to 78%, and 8 to 78% (Figure 5C). ROC curve analysis yielded area under curves (AUCs) of 0.885, 0.878, and 0.879 for predicting AP in PSD patients in the training, validation, and overall cohorts, respectively (Figure 5D).

### 3.7 External validation of nomogram model

A retrospective study collected 500 stroke cases with ≥2 on the water drinking test from Jinshan District's health centers (2020.01-2023.10) as an external validation cohort (297 males, 203 females). Among them, 112 (22.40%) had AP, and 388 (77.60%) did not. Clinical data are in Supplementary Table 6, and lab tests are in Supplementary Table 7.

**Figure 6:**
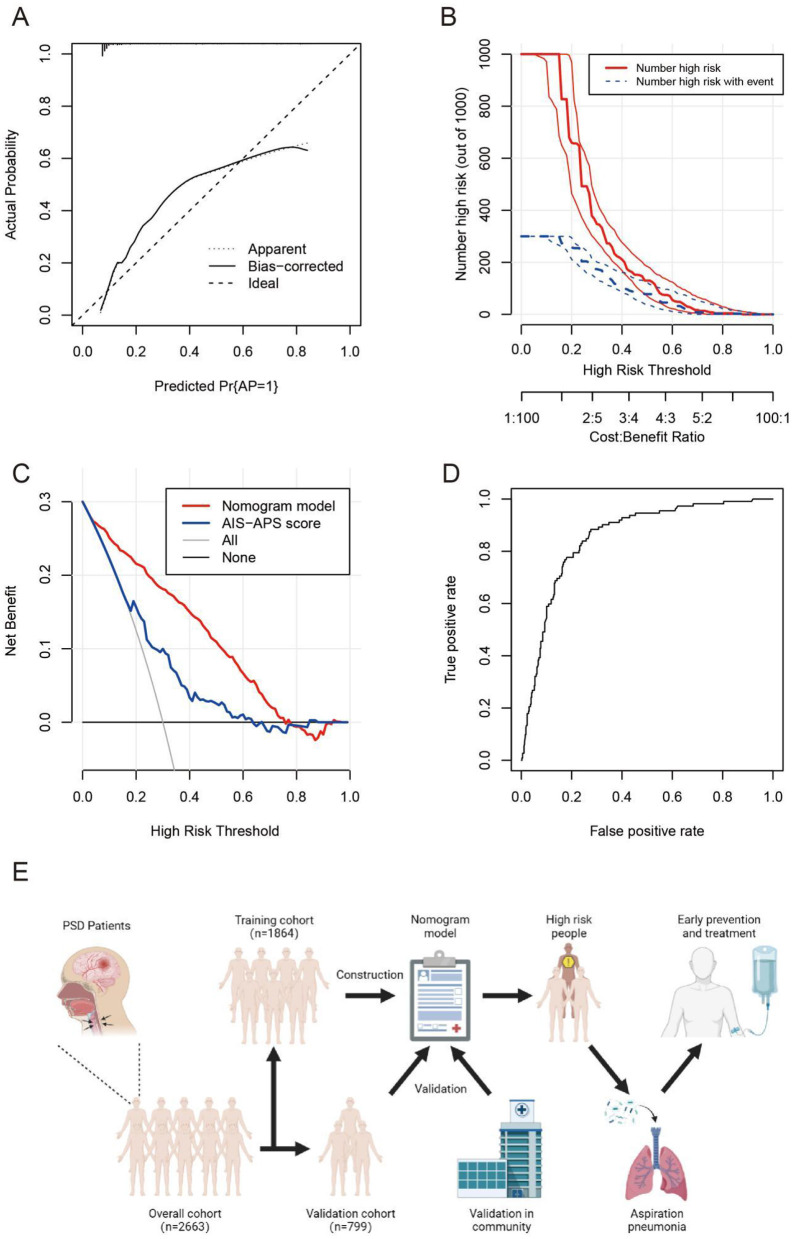
The calibration curve **(A)**, clinical impact curve **(B)**, clinical decision curve **(C)**, and ROC curve **(D)** in the external validation cohort of the nomogram. The basic process of constructing and validating the nomogram prediction model **(E)**. AP, aspiration pneumonia; AIS-APS score, acute ischemic stroke-associated pneumonia score.

The nomogram model demonstrated strong consistency in external validation with a C-index of 0.855 (95% CI: 0.783–0.927) after 1,000 bootstrap resamplings. Calibration curve analysis confirmed the model's effectiveness and reliability, showing good agreement between predicted and observed AP incidence among PSD patients (Figure 6A). The clinical impact curve indicated high concordance in identifying high-risk AP patients when the predicted probability exceeded 20% (Figure 6B). Clinical decision analysis revealed a net benefit for patients within a threshold probability range of 5–75% (Figure 6C). ROC analysis yielded an AUC of 0.855, sensitivity of 0.724, specificity of 0.884, and Youden's index of 0.608 for predicting AP in stroke patients (Figure 6D). The basic process of constructing and validating the nomogram prediction model for AP occurrence in PSD patients in this study is detailed in Figure 6E.

## 4 Discussion

### 4.1 Epidemiological characteristics of AP in patients with PSD

PSD, a common stroke complication with an incidence over 50%, significantly impacts patient rehabilitation. AP, which poses a severe risk to PSD patients, occurs in 5–15% of stroke patients but up to 30–50% in PSD patients (24). In this study, we found PSD patients had a higher AP probability (21.70%) than stroke patients without dysphagia (5.05%). Despite variations in incidence, healthcare professionals still must prioritize early intervention and prevention for PSD patients (25, 26).

### 4.2 Risk factors for AP occurrence in PSD

Our study identified several independent factors that may contribute to the risk of AP in PSD patients. Based on existing research, we analyze each variable's impact on AP development in PSD patients:

(1) Advanced age constitutes a significant independent risk factor for AP in PSD patients, mediated through multiple pathways including increased comorbidity burden, immunosenescence, and diminished pulmonary defense mechanisms (27, 28). This risk is further compounded by the severity of dysphagia and delayed recovery of swallowing function (29, 30). While the independent contribution of age remains debated in some studies, emerging evidence highlights the particular significance of recent swallowing deterioration (within 3 months) as a strong predictor (OR = 3.584) (31). Additional age-related factors such as alterations in oral microbiota composition and progressive pharyngeal muscle atrophy have been implicated in the pathogenesis of AP (32, 33).
(2) A NIHSS score ≥10.5 demonstrates significant predictive value for both aspiration pneumonia (AP) and dysphagia in stroke patients, serving as a quantitative indicator of stroke severity and associated swallowing dysfunction (34, 35). Specific NIHSS components—particularly facial palsy, limb weakness, and impaired consciousness—independently contribute to elevated AP risk by compromising airway protection mechanisms (36). These findings establish the NIHSS as an essential clinical tool for comprehensive evaluation of post-stroke swallowing impairment and subsequent AP risk stratification (37).
(3) Emerging evidence suggests hyperlipidemia may paradoxically confer reduced AP risk, potentially mediated through the immunomodulatory effects of long-term statin therapy (38, 39). However, abnormal lipid profiles have been associated with increased pneumonia susceptibility, including worse corona virus disease 2019 (COVID-19) outcomes (40). This underscores the importance of rigorous metabolic management for obesity and dyslipidemia in preventing severe post-vaccination COVID-19 complications (41). Notably, genetic factors such as ApoE polymorphisms appear to differentially influence AP risk, demonstrating significant association in younger stroke patients but not elderly populations (42).
(4) Hyperhomocysteinemia significantly elevates aspiration pneumonia (AP) risk through multiple pathological mechanisms. It induces neuronal damage, chronic inflammation, and immune dysfunction, collectively impairing swallowing function . The condition promotes excessive release of pro-inflammatory mediators [Interleukin- 6 (IL-6), tumor necrosis factor-α (TNF-α)], which compromise laryngopharyngeal protective reflexes (43, 44). Furthermore, its well-established role in atherosclerosis pathogenesis contributes to stroke occurrence and subsequent dysphagia exacerbation (45, 46). This risk is particularly pronounced in stroke patients with hyperhomocysteinemia, as severe vascular endothelial dysfunction further potentiates swallowing impairment (47).
(5) Cardiac insufficiency significantly increases AP risk in PSD patients through multiple pathophysiological mechanisms. Primarily, it induces pulmonary congestion and edema, impairing respiratory function and creating favorable conditions for bacterial proliferation (48, 49). The condition also promotes gastrointestinal edema, which elevates aspiration susceptibility (50), while concurrently impairing mucociliary clearance mechanisms (51). These patients frequently exhibit compounded vulnerabilities including immunocompromised status and malnutrition, which collectively diminish host defenses (52). Cardiovascular dysfunction further exacerbates AP risk by impairing immune competence and bacterial clearance capacity (53). Concomitant digestive impairments, including poor appetite and malabsorption, create a malnutrition-inflammation cycle that substantially compromises pulmonary defenses (54). Notably, the severity of cardiac insufficiency correlates with both stroke prognosis and AP incidence, suggesting a bidirectional relationship between cardiovascular and neurological outcomes in this patient population (55).
(6) Elevated inflammatory markers—including CRP, WBC count, and NE%—serve as clinically significant indicators of systemic inflammation and immune activation that substantially increase AP risk (56–58). As a sensitive acute-phase reactant, CRP levels directly correlate with inflammatory burden (59). Notably, stroke patients with dysphagia demonstrating CRP levels >10 mg/L exhibit a 97% increased AP risk (60), primarily mediated through dysphagia-related infectious complications (61). Mechanistically, CRP modulates pulmonary inflammatory responses and immune cell activity, thereby creating a permissive environment for AP development (62). Similarly, elevated WBC counts and neutrophilia reflect both inflammatory intensity and infection severity, providing additional prognostic value for AP risk stratification.
(7) Hemoglobin levels serve as a sensitive biomarker of nutritional status, with decreased values indicating malnutrition—a well-established risk factor for pneumonia, particularly among elderly populations, and a significant prognostic indicator when coexisting with comorbidities (63). As the primary oxygen transport molecule, hemoglobin deficiency induces tissue hypoxia, potentially exacerbating pulmonary compromise. Clinical evidence suggests that early red blood cell transfusion may reduce pneumonia-associated mortality in hospitalized patients (64). Epidemiological studies demonstrate an inverse correlation between hemoglobin levels and pneumonia risk, with each 1 g/L increase corresponding to a 2% risk reduction (65). Notably, emerging research indicates that upregulated hemoglobin metabolism may represent a protective leukocyte response mechanism during infection (66).
(8) Prealbumin serves as a dual biomarker, reflecting both nutritional status and systemic inflammation. In stroke patients, decreased serum prealbumin levels during hospitalization are strongly predictive of infection severity and clinical prognosis, providing valuable guidance for therapeutic decision-making (67). Its utility in early acute-phase detection enables timely identification of stroke-associated pneumonia risk, particularly in ischemic stroke patients, facilitating prompt clinical interventions (68). Notably, among elderly stroke patients with pulmonary infections, prealbumin levels demonstrate an inverse correlation with infection severity, further underscoring its prognostic value (69).
(9) Fasting blood glucose serves as a routinely measured and clinically valuable biomarker (70). Elevated admission glucose levels demonstrate differential predictive value for ICU admission risk in community-acquired pneumonia (CAP) patients, showing significant association in non-diabetics (OR = 1.25) but minimal effect in diabetics (OR = 1.05) after adjustment for disease severity and comorbidities (71). Patients with diabetes mellitus face compounded risks for swallowing dysfunction and aspiration pneumonia due to metabolic dysregulation and immune impairment. This is substantiated by findings of significantly higher prevalence of esophageal motility disorders in diabetic populations (60% vs. 29.6% in non-diabetics) (72), highlighting the critical need for rigorous glycemic control and comprehensive management in this vulnerable patient group.
(10) Elevated BNP levels and serum sodium abnormalities serve as important biomarkers of cardiopulmonary dysfunction that significantly increase aspiration pneumonia (AP) risk (73). Pathophysiologically, increased BNP concentrations indicate cardiovascular stress that compromises both immune competence and pulmonary defense mechanisms (74). Concurrently, serum sodium dysregulation contributes to AP pathogenesis through dual mechanisms: (1) blood volume alterations impairing mucociliary clearance, and (2) direct compromise of airway protective reflexes (75). These findings underscore the critical interplay between cardiovascular homeostasis and respiratory vulnerability in AP development.
(11) Analysis of critical factors: while previous studies have identified smoking and chronic obstructive pulmonary disease (COPD) as independent risk factors for AP in PSD patients (76, 77), our current analysis did not replicate these associations. This discrepancy may be attributed to several contemporary factors: (1) significant reductions in smoking prevalence due to effective public health campaigns and tobacco control policies (78); (2) improved air quality and medical management leading to decreased COPD incidence (79); and (3) potential underreporting of these risk factors during routine clinical history-taking.

Nevertheless, the established pathophysiological mechanisms remain valid: smoking induces respiratory mucosal damage, impairs mucociliary clearance, and compromises pulmonary immunity, thereby increasing AP susceptibility in dysphagic stroke patients (80, 81). Similarly, COPD patients exhibit chronic airway inflammation, reduced pulmonary function, and excessive mucus production—all of which synergize with dysphagia to substantially elevate post-stroke AP risk (82, 83). The coexistence of dysphagia and COPD appears particularly detrimental to airway protective mechanisms (84).

### 4.3 The importance and clinical value of constructing a nomogram prediction model of AP for PSD patients

As machine learning evolve, diagnostic and prognostic models utilizing biomarkers and clinical features have proliferated in medicine (85). However, models accurately predicting AP in PSD patients are scarce. Hence, developing a precise risk prediction nomogram is crucial for identifying high-risk PSD patients prone to AP.

Our study used multivariable Logistic regression to quantify factors influencing AP in PSD, creating a nomogram scoring system for healthcare providers. This system assigns scores to clinical and lab factors, enabling risk assessment (18). Our nomogram identifies high-risk patients by integrating variables such as hyperglycemia, malnutrition (low PA), cardiac dysfunction (elevated BNP), and systemic inflammation (elevated CRP/WBC). For these individuals, tailored interventions may mitigate AP risk and sequelae: (1) Optimizing Swallowing Function & Nutritional Support: Early dysphagia screening (e.g., bedside swallowing tests or videofluoroscopy) (86) and modified diets (thickened liquids, postural adjustments) can reduce aspiration risk (87). Malnourished patients (low PA) may benefit from enteral nutrition protocols with close monitoring of aspiration risk (88). (2) Glycemic & Metabolic Control: Strict management of hyperglycemia (FBG) and hyperlipidemia may attenuate systemic inflammation and infection susceptibility (89). (3) Cardiopulmonary Management: Aggressive treatment of cardiac insufficiency (e.g., diuretics for volume overload, guideline-directed heart failure therapy) and early mobilization could reduce pulmonary congestion and aspiration-related complications (90). (4) Infection Prevention: Prophylactic strategies (oral hygiene, probiotics, or selective digestive decontamination in high-risk cases) may lower bacterial load in aspirated material (91). (5) Biomarker-Guided Monitoring: Serial measurements of CRP, BNP, and WBC could help detect subclinical infections or worsening cardiac function, prompting preemptive interventions.

The nomogram informs medical institutions and policymakers about PSD-related AP trends, aiding in crafting effective prevention strategies. Future studies should validate whether targeting these modifiable factors (e.g., via bundled care protocols) reduces AP incidence in high-risk cohorts. Moreover, integrating the prediction model into an electronic health monitoring system to provide automated alerts for high-risk patients could effectively improve the sensitivity of AP screening and reduce the clinical burden on physicians.

### 4.4 Limitation

This study has limitations: retrospective data may introduce information bias, and we couldn't explore all factors due to many clinical variables. Moreover, our results may be region- and population-specific, limiting generalization.

## 5 Conclusion

In conclusion, our study demonstrates that PSD patients face significantly elevated AP risk, necessitating early intervention. We identified age, NIHSS score, hyperlipidemia, hyperhomocysteinemia, cardiac insufficiency, CRP, WBC, NE%, Hb, FBG, PA, BNP and Na+ as key predictors, which were incorporated into a clinically practical nomogram for individualized AP risk prediction. This tool enables clinicians to proactively identify high-risk patients for targeted interventions (e.g., modified diets, respiratory therapy) while optimizing resource allocation in both hospital and community settings. While our findings provide actionable insights for PSD management, future studies should focus on external validation of the model and investigation of additional risk factors (e.g., microbiome, immunologic profiles) across diverse populations to further refine predictive accuracy.

## Data Availability

The raw data supporting the conclusions of this article will be made available by the authors, without undue reservation.
