# Supplementary material for: Innovative nomogram for predictive risk stratification of aspiration pneumonia in post-stroke dysphagia patients
Source: Front Neurol. 2025 Jun 3;16:1556541. doi: 10.3389/fneur.2025.1556541 (PMC12170325; doi:10.3389/fneur.2025.1556541)
Supplement: Supplementary file 1 [file Table_1.docx]

**Supplementary Table 1** Comparison of clinical characteristics of patients with acute stroke

| **Clinical characters** | **Acute stroke patients (n=7134)** | | ***t or x^2^*** | ***P*** |
| --- | --- | --- | --- | --- |
|  | **Non-AP (n=6330)** | **AP (n=804)** |  |  |
| **Gender (n, %)** |  |  | 1.769 | 0.184 |
| **Male** | 3831 (60.5) | 467 (58.1) |  |  |
| **Female** | 2499 (39.5) | 337 (41.9) |  |  |
| **Age [Years, M (P25, P75)]** | 68 (60, 77) | 76 (66, 83) | 11.990 | 0.000 |
| **Hospital stay [Days, M (P25, P75)]** | 10 (8, 13) | 15 (9, 26) | 17.470 | 0.000 |
| **Discharge Status** |  |  | 725.349 | 0.000 |
| **Improved (n, %)** | 5895 (93.1) | 502 (62.4) |  |  |
| **Death or not improved (n, %)** | 435 (6.9) | 302 (37.6) |  |  |
| **Type of stroke (n, %)** |  |  | 63.556 | 0.000 |
| **Ischemic stroke** | 5149 (81.3) | 558 (69.4) |  |  |
| **Hemorrhagic stroke** | 1181 (18.7) | 246 (30.6) |  |  |
| **Water drinking test score [Points, M (P25, P75)]** | 1 (1, 2) | 5 (1, 5) | 47.200 | 0.000 |
| **NIHSS score [Points, M (P25, P75)]** | 2 (0, 3) | 3 (2, 6) | 190.600 | 0.000 |
| **Smoking history (n, %)** | 371 (5.9) | 49 (6.1) | 0.070 | 0.791 |
| **Drinking history (n, %)** | 256 (4.0) | 30 (3.7) | 0.181 | 0.670 |
| **BMI [kg/m^2^, M (P25, P75)]** | 24.0 (22.7, 25.5) | 24.0 (22.3, 24.8) | 2.152 | 0.314 |
| **Underlying diseases (n, %)** |  |  |  |  |
| **Encephalatrophy** | 1269 (20.0) | 120 (14.9) | 11.937 | 0.001 |
| **Hypertension** | 4772 (75.4) | 606 (75.4) | 0.000 | 0.993 |
| **Diabetes** | 2035 (32.1) | 240 (29.9) | 1.734 | 0.188 |
| **Hyperlipidemia** | 1367 (21.6) | 66 (8.2) | 79.641 | 0.000 |
| **Hyperuricemia** | 293 (4.6) | 24 (3.0) | 4.539 | 0.033 |
| **Hyperhomocysteinemia** | 1342 (21.2) | 99 (12.3) | 34.956 | 0.000 |
| **Coronary heart disease** | 570 (9.0) | 149 (18.5) | 71.455 | 0.000 |
| **Valvular disease** | 346 (5.5) | 33 (4.1) | 2.629 | 0.105 |
| **Atrial fibrillation** | 618 (9.8) | 218 (27.1) | 207.612 | 0.000 |
| **Cardiac insufficiency** | 325 (5.1) | 168 (20.9) | 275.482 | 0.000 |
| **Pulmonary underlying diseases** | 368 (5.8) | 78 (9.7) | 18.399 | 0.000 |
| **Hepatic insufficiency** | 395 (6.2) | 108 (13.4) | 56.316 | 0.000 |
| **Renal insufficiency** | 291 (4.6) | 92 (11.4) | 65.804 | 0.000 |
| **Malignant tumors history** | 216 (3.4) | 33 (4.1) | 1.015 | 0.314 |

Abbreviation: AP, aspiration pneumonia; NIHSS, national institute of health stroke scale; BMI, body mass index.
