# Supplementary material for: Innovative nomogram for predictive risk stratification of aspiration pneumonia in post-stroke dysphagia patients
Source: Front Neurol. 2025 Jun 3;16:1556541. doi: 10.3389/fneur.2025.1556541 (PMC12170325; doi:10.3389/fneur.2025.1556541)
Supplement: Supplementary file 2 [file Table_2.docx]

**Supplementrary Table 2** The assignment details of clinical characters and labortory examinations

| **Variables** | **Factors** | **Assignment situation** |
| --- | --- | --- |
| ***X1*** | Age | ≤59=0; 60-75=1; ＞75=2 |
| ***X2*** | Type of stroke | Ischemic stroke=0; Hemorrhagic stroke=1 |
| ***X3*** | NIHSS score | ≤5=0; 6-20=1; ＞20=2 |
| ***X4*** | Encephalatrophy | No=0; Yes=1 |
| ***X5*** | Diabetes | No=0; Yes=1 |
| ***X6*** | Hyperlipidemia | No=0; Yes=1 |
| ***X7*** | Hyperuricemia | No=0; Yes=1 |
| ***X8*** | Hyperhomocysteinemia | No=0; Yes=1 |
| ***X9*** | Coronary heart disease | No=0; Yes=1 |
| ***X10*** | Atrial fibrillation | No=0; Yes=1 |
| ***X11*** | Cardiac insufficiency | No=0; Yes=1 |
| ***X12*** | Pulmonary underlying diseases | No=0; Yes=1 |
| ***X13*** | Hepatic insufficiency | No=0; Yes=1 |
| ***X14*** | Renal insufficiency | No=0; Yes=1 |
| ***X15*** | CRP | ≤10=0; (10, 20]=1; (20, 30]=2; (30, 40]=3; (40, 50]=4; ＞50=5 |
| ***X16*** | WBC | ≤10=0; ＞10=1 |
| ***X17*** | NE% | ≤80=0; ＞80=1 |
| ***X18*** | LY% | ＞20=0; ≤20=1 |
| ***X19*** | NE | ≤7.5=0; ＞7.5=1 |
| ***X20*** | LY | ＞1=0; ≤1=1 |
| ***X21*** | NE/LY | ≤3=0; ＞3=1 |
| ***X22*** | RBC | ＞5.0=0; ≤5.0=1 |
| ***X23*** | Hb | ＞120=0; (90, 120]=1; (60, 90]=2; ≤60=3 |
| ***X24*** | Hct | ＞0.35=0; ≤0.35=1 |
| ***X25*** | Plt | ＞100=0; ≤100=1 |
| ***X26*** | WBC/RBC | ≤4=0; ＞4=1 |
| ***X27*** | FBG | 4-7=0; ＜4 or ＞7=1 |
| ***X28*** | TC | ≤5.2=0; ＞5.2=1 |
| ***X29*** | TG | 0.56-1.7=0; ＜0.56 or ＞1.7=1 |
| ***X30*** | LDL | ≤3.4=0; ＞3.4=1 |
| ***X31*** | ALT | ≤40=0; ＞40=1 |
| ***X32*** | TP | ＞60=0; ≤60=1 |
| ***X33*** | Alb | ＞40=0; 30-40=1; ≤30=2 |
| ***X34*** | PA | ＞180=0; (150, 180]=1; (100, 150]=2; ≤100=3 |
| ***X35*** | Scr | ≤133=0; ＞133=1 |
| ***X36*** | GFR | ＞90=0; (60, 90]=1; (30, 60]=2; (15, 30]=3; ≤15=4 |
| ***X37*** | BNP | ≤100=0; (100, 400]=1; ＞400=2 |
| ***X38*** | K^+^ | 3.5-5.0=0; ＜3.5 or ＞5.0=1 |
| ***X39*** | Na^+^ | 135-145=0; ＜135 or ＞145=1 |
| ***Y*** | AP | No=0; Yes=1 |
