# Supplementary material for: Innovative nomogram for predictive risk stratification of aspiration pneumonia in post-stroke dysphagia patients
Source: Front Neurol. 2025 Jun 3;16:1556541. doi: 10.3389/fneur.2025.1556541 (PMC12170325; doi:10.3389/fneur.2025.1556541)
Supplement: Supplementary file 3 [file Table_3.docx]

**Supplementary Table 3 Comparison of general clinical data between Non-AP and AP group PSD patients**

| **Factors** | **Non-AP (n=2085)** | **AP (n=578)** | **Total (n=2663)** | **t or x^2^** | ***P*** |
| --- | --- | --- | --- | --- | --- |
| **Gender (n, %)** |  |  |  |  |  |
| **Female** | 858 (41.15) | 235 (40.66) | 1093 (41.04) | 0.046 | 0.831 |
| **Male** | 1227 (58.85) | 343 (59.34) | 1570 (58.96) |  |  |
| **Age [Years, M(P25, P75)]** | 68 (59, 77) | 75 (64, 82) | 69 (60, 79) | 7.506 | ＜0.001 |
| **Length of hospital stay [Days, M(P25, P75)]** | 11 (7, 15) | 16 (9, 29) | 11 (8, 16) | 9.191 | ＜0.001 |
| **Way of discharge (n, %)** |  |  |  | 278.507 | ＜0.001 |
| **Improved** | 1807 (86.67) | 319 (55.19) | 2126 (79.83) |  |  |
| **Death or non recovery** | 278 (13.33) | 259 (44.81) | 537 (20.17) |  |  |
| **Type of stroke (n, %)** |  |  |  | 4.873 | 0.027 |
| **Ischemic stroke** | 1596 (76.55) | 359 (62.11) | 1955 (73.41) |  |  |
| **Hemorrhagic stroke** | 489 (23.45) | 219 (37.89) | 708 (26.59) |  |  |
| **NIHSS score [Points, M (P25, P75)]** | 3 (0, 3) | 3 (2, 6) | 3 (1, 3) | 11.760 | ＜0.001 |
| **Smoking history (n, %)** | 164 (7.87) | 42 (7.27) | 206 (7.74) | 0.228 | 0.633 |
| **Drinking history (n, %)** | 114 (5.47) | 26 (4.50) | 140 (5.26) | 0.854 | 0.356 |
| **BMI [kg/m2, M (P25, P75)]** | 24 (22.8, 25.6) | 24 (22.5, 25.0) | 24 (22.8, 25.4) | 1.012 | 0.312 |
| **Vital signs (n, %)** |  |  |  |  |  |
| **Body temperature (℃)** |  |  |  | 22.513 | ＜0.001 |
| **36-37** | 1851 (88.78) | 470 (81.31) | 2321 (87.16) |  |  |
| **＜36 or ＞37** | 234 (11.22) | 108 (18.69) | 342 (12.84) |  |  |
| **Pulse (Per min)** |  |  |  | 11.837 | ＜0.001 |
| **60-100** | 2012 (96.50) | 539 (93.25) | 2551 (95.79) |  |  |
| **＜60 or ＞100** | 73 (3.50) | 39 (6.75) | 112 (4.21) |  |  |
| **Breathe (Per min)** |  |  |  | 112.747 | ＜0.001 |
| **12-20** | 1863 (89.35) | 415 (71.80) | 2278 (85.54) |  |  |
| **＜12 or ＞20** | 222 (10.65) | 163 (28.20) | 385 (14.46) |  |  |
| **Blood pressure (mmHg)** |  |  |  | 0.034 | 0.853 |
| **Systolic pressure＜140 and diastolic pressure＜90** | 636 (30.50) | 174 (30.10) | 810 (30.42) |  |  |
| **Systolic pressure≥140 or diastolic pressure≥90** | 1449 (69.50) | 404 (69.90) | 1853 (69.58) |  |  |
| **Underlying disease (n, %)** |  |  |  |  |  |
| **Encephalatrophy** | 344 (16.50) | 72 (12.46) | 416 (15.62) | 5.609 | 0.018 |
| **Hypertension** | 530 (25.42) | 150 (25.95) | 680 (25.54) | 0.067 | 0.795 |
| **Diabetes** | 629 (30.17) | 150 (25.95) | 779 (29.25) | 3.887 | 0.049 |
| **Hyperlipidemia** | 370 (17.75) | 39 (6.75) | 409 (15.36) | 42.110 | ＜0.001 |
| **Hyperuricemia** | 90 (4.32) | 12 (2.08) | 102 (3.83) | 6.167 | 0.013 |
| **Hyperhomocysteinemia** | 496 (23.79) | 153 (26.47) | 649 (24.37) | 1.766 | 0.018 |
| **Coronary heart disease** | 211 (10.12) | 110 (19.03) | 321 (12.05) | 33.899 | ＜0.001 |
| **Atrial fibrillation** | 244 (11.70) | 145 (25.09) | 389 (14.61) | 64.987 | ＜0.001 |
| **Cardiac insufficiency** | 136 (6.52) | 127 (21.97) | 263 (9.88) | 121.358 | ＜0.001 |
| **Pulmonary underlying diseases** | 125 (6.00) | 42 (7.27) | 167 (6.27) | 1.244 | 0.265 |
| **Hepatic insufficiency** | 129 (6.19) | 71 (12.28) | 200 (7.51) | 24.216 | ＜0.001 |
| **Renal insufficiency** | 107 (5.13) | 62 (10.73) | 169 (6.35) | 23.833 | ＜0.001 |
| **Malignant tumors history** | 74 (3.55) | 23 (3.98) | 97 (3.64) | 0.238 | 0.625 |
