# Supplementary material for: Innovative nomogram for predictive risk stratification of aspiration pneumonia in post-stroke dysphagia patients
Source: Front Neurol. 2025 Jun 3;16:1556541. doi: 10.3389/fneur.2025.1556541 (PMC12170325; doi:10.3389/fneur.2025.1556541)
Supplement: Supplementary file 4 [file Table_4.docx]

**Supplementary Table 4 Comparison of important laboratory examinations between Non-AP group and AP group PSD patients**

| **Factor** | **Non-AP**  **n=2085** | **AP**  **n=578** | **Total**  **n=2663** | ***t*** | ***P*** |
| --- | --- | --- | --- | --- | --- |
| **CRP [mg/L, M (P25, P75)]** | 2.29 (0.00, 22.40) | 82.48 (39.95, 142.80) | 5.53 (0.00, 38.00) | 16.100 | ＜0.001 |
| **Routine blood test [M (P25, P75)]** |  |  |  |  |  |
| **WBC (×10^9^/L)** | 7.5 (5.9, 9.8) | 13.0 (9.7, 17.2) | 8.1 (6.2, 11.7) | 69.430 | ＜0.001 |
| **NE% (%)** | 68.8 (61.5, 80.0) | 87.8 (81.9, 91.4) | 73.7 (63.8, 86.4) | 19.740 | ＜0.001 |
| **LY% (%)** | 26.7 (20.1, 32.3) | 18.1 (13.0, 25.5) | 25.1 (17.8, 31.1) | 9.369 | ＜0.001 |
| **NE (×10^9^/L)** | 5.11 (3.62, 7.44) | 11.16 (7.87, 14.80) | 5.78 (3.87, 9.70) | 82.530 | ＜0.001 |
| **LY (×10^9^/L)** | 1.66 (1.27, 2.06) | 1.47 (1.06, 1.96) | 1.62 (1.23, 2.04) | 3.673 | ＜0.001 |
| **NE/LY** | 3.14 (2.07, 5.12) | 7.37 (5.11, 10.53) | 3.60 (2.28, 6.68) | 20.980 | ＜0.001 |
| **RBC (×10^12^/L)** | 4.10 (3.75, 4.51) | 3.57 (2.90, 4.10) | 4.06 (3.58, 4.44) | 17.830 | ＜0.001 |
| **Hb (g/L)** | 125 (114, 137) | 109 (88, 125) | 124 (109, 135) | 13.450 | ＜0.001 |
| **Hct (L/L)** | 0.37 (0.34, 0.40) | 0.33 (0.27, 0.37) | 0.37 (0.33, 0.40) | 16.710 | ＜0.001 |
| **Plt (×10^9^/L)** | 186 (145, 223) | 146 (101, 187) | 177 (134, 218) | 10.880 | ＜0.001 |
| **WBC/RBC (×10^-3^)** | 1.78 (1.39, 2.43) | 3.62 (2.66, 5.45) | 1.97 (1.48, 3.09) | 24.200 | ＜0.001 |
| **FBG (mmol/L)** | 6.65 (5.33, 7.71) | 7.61 (7.01, 11.78) | 7.13 (5.49, 8.49) | 15.060 | ＜0.001 |
| **GHb (%)** | 5.82 (5.80, 6.90) | 5.82 (5.80, 6.78) | 5.82 (5.80, 6.90) | 0.447 | 0.655 |
| **Blood fat [M (P25, P75)]** |  |  |  |  |  |
| **TC (mmol/L)** | 4.21 (3.73, 5.07) | 4.18 (3.49, 4.78) | 4.18 (3.69, 5.01) | 4.485 | ＜0.001 |
| **TG (mmol/L)** | 1.36 (0.95, 1.71) | 1.27 (0.89, 1.45) | 1.33 (0.94, 1.67) | 2.329 | 0.020 |
| **LDL (mmol/L)** | 2.85 (2.28, 3.34) | 2.81 (2.14, 3.01) | 2.85 (2.24, 3.27) | 4.194 | ＜0.001 |
| **HDL (mmol/L)** | 1.08 (0.92, 1.23) | 1.08 (0.92, 1.25) | 1.08 (0.92, 1.23) | 0.469 | 0.639 |
| **Hepatic function [M (P25, P75)]** |  |  |  |  |  |
| **ALT (U/L)** | 25.00 (16.00, 33.19) | 33.19 (20.00, 52.00) | 27.00 (16.00, 33.19) | 9.551 | ＜0.001 |
| **TP (g/L)** | 62 (60, 65) | 62 (55, 63) | 62 (59, 65) | 11.710 | ＜0.001 |
| **Alb (g/L)** | 35.48 (34.00, 38.00) | 33.00 (28.00, 35.48) | 35.48 (33.00, 37.00) | 17.830 | ＜0.001 |
| **PA (mg/L)** | 210.86 (181.00, 243.00) | 154.00 (105.25, 210.86) | 210.86 (159.50, 232.00) | 19.240 | ＜0.001 |
| **Renal function [M (P25, P75)]** |  |  |  |  |  |
| **Scr (umol/L)** | 78.00 (63.00, 84.41) | 84.41 (66.25, 101.75) | 80.00 (64.00, 85.00) | 6.024 | ＜0.001 |
| **GFR [mL/ (min×1.73m^2^)]** | 99.64 (62.52, 113.30) | 62.52 (62.52, 113.30) | 94.07 (62.52, 113.30) | 6.352 | ＜0.001 |
| **BNP (ng/L)** | 86.91 (25.46, 145.78) | 198.00 (78.48, 519.97) | 118.00 (31.17, 168.30) | 14.770 | ＜0.001 |
| **Electrolyte [M (P25, P75)]** |  |  |  |  |  |
| **K^+^ (mmol/L)** | 3.94 (3.73, 4.13) | 4.20 (3.94, 4.60) | 3.94 (3.77, 4.22) | 15.150 | ＜0.001 |
| **Na^+^ (mmol/L)** | 143 (141, 144) | 145 (142, 152) | 143 (141, 145) | 15.640 | ＜0.001 |
