# Supplementary material for: Innovative nomogram for predictive risk stratification of aspiration pneumonia in post-stroke dysphagia patients
Source: Front Neurol. 2025 Jun 3;16:1556541. doi: 10.3389/fneur.2025.1556541 (PMC12170325; doi:10.3389/fneur.2025.1556541)
Supplement: Supplementary file 5 [file Table_5.docx]

**Supplementary Table 5** Comparison of baseline data between training group and validation group.

| **Factors** | **Training group** | | | |  | **Validation group** | | | | ***P^#^*** |
| --- | --- | --- | --- | --- | --- | --- | --- | --- | --- | --- |
|  | **Total**  **(n = 1864)** | **Non-AP**  **(n = 1462)** | **AP**  **(n = 402)** | ***P^$^*** |  | **Total**  **(n=799)** | **Non-Ap**  **(n=623)** | **AP**  **(n=176)** | ***P^&^*** |  |
| **Gender (n, %)** |  |  |  |  |  |  |  |  |  |  |
| **Male** | 1092 (58.58) | 856 (58.55) | 236 (58.71) | 0.955 |  | 478 (59.82) | 371 (59.55) | 107 (60.80) | 0.766 | 0.661 |
| **Female** | 772 (41.42) | 606 (41.45) | 166 (41.29) |  |  | 321 (40.18) | 252 (40.45) | 69 (39.20) |  |  |
| **Age [Years, M(P25, P75)]** | 69 (60, 79) | 68 (59, 77) | 75 (64, 83) | ＜0.001 |  | 70 (60, 79) | 68 (58, 78) | 75 (65, 82) | ＜0.001 | 0.983 |
| **Age (Years, %)** |  |  |  |  |  |  |  |  |  |  |
| **≤59** | 452 (24.25) | 377 (25.79) | 75 (18.66) | ＜0.001 |  | 195 (24.41) | 166 (26.65) | 29 (16.48) | ＜0.001 | 0.334 |
| **60-74** | 755 (40.50) | 631 (43.16) | 124 (30.84) |  |  | 305 (38.17) | 251 (40.29) | 54 (30.68) |  |  |
| **≥75** | 657 (35.25) | 454 (31.05) | 203 (50.50) |  |  | 299 (37.42) | 206 (33.07) | 93 (52.84) |  |  |
| **Length of hospital stay [Days, M (P25, P75)]** | 11 (8, 17) | 11 (8, 15) | 16 (9, 30) | ＜0.001 |  | 11 (7, 16) | 10 (7, 14) | 16 (10, 28) | ＜0.001 | 0.252 |
| **Way of discharge (n, %)** |  |  |  |  |  |  |  |  |  |  |
| **Improved** | 1488 (79.83) | 1266 (86.59) | 222 (55.22) | ＜0.001 |  | 638 (79.85) | 541 (86.84) | 97 (55.11) | ＜0.001 | 0.990 |
| **Death or non recovery** | 376 (20.17) | 196 (13.41) | 180 (44.78) |  |  | 161 (20.15) | 82 (13.16) | 79 (44.89) |  |  |
| **Type of stroke (n, %)** |  |  |  |  |  |  |  |  |  |  |
| **Ischemic stroke** | 1200 (64.38) | 960 (65.66) | 240 (59.70) | 0.027 |  | 491 (61.45) | 381 (61.16) | 110 (62.50) | 0.756 | 0.151 |
| **Hemorrhagic stroke** | 664 (35.62) | 502 (34.34) | 162 (40.30) |  |  | 308 (38.55) | 242 (38.84) | 66 (37.50) |  |  |
| **NIHSS score [n, M (P25, P75)]** | 3 (1, 3) | 3 (0, 3) | 3 (3, 6) | ＜0.001 |  | 3 (1, 3) | 3 (0, 3) | 3 (2, 3) | ＜0.001 | 0.161 |
| **NIHSS score (n, %)** |  |  |  |  |  |  |  |  |  |  |
| **≤5** | 1571 (84.28) | 1282 (87.69) | 289 (71.89) | ＜0.001 |  | 683 (85.48) | 545 (87.48) | 138 (78.41) | 0.005 | 0.367 |
| **6-20** | 240 (12.88) | 156 (10.67) | 84 (20.90) |  |  | 103 (12.89) | 71 (11.40) | 32 (18.18) |  |  |
| **＞20** | 53 (2.84) | 24 (1.64) | 29 (7.21) |  |  | 13 (1.63) | 7 (1.12) | 6 (3.41) |  |  |
| **Smoking history (n, %)** | 140 (7.51) | 112 (7.66) | 28 (6.97) | 0.639 |  | 66 (8.26) | 52 (8.35) | 14 (7.95) | 0.868 | 0.507 |
| **Drinking history (n, %)** | 91 (4.88) | 72 (4.92) | 19 (4.73) | 0.870 |  | 49 (6.13) | 42 (6.74) | 7 (3.89) | 0.177 | 0.230 |
| **BMI [kg/m^2^, M (P25, P75)]** | 24 (22.7, 25.4) | 24 (22.7, 25.4) | 24 (22.3, 25.4) | 0.860 |  | 24 (22.9, 25.5) | 24 (22.9, 25.7) | 24 (22.6, 24.5) | 0.043 | 0.620 |
| **Encephalatrophy (n, %)** | 281 (15.07) | 234 (16.01) | 47 (11.69) | 0.032 |  | 135 (16.90) | 110 (17.66) | 25 (14.20) | 0.028 | 0.236 |
| **Hypertension (n, %)** | 1369 (73.44) | 1090 (74.56) | 279 (69.40) | 0.038 |  | 614 (76.85) | 465 (74.64) | 149 (84.66) | 0.005 | 0.065 |
| **Diabetes (n, %)** | 545 (29.24) | 444 (30.37) | 101 (25.12) | 0.041 |  | 234 (29.29) | 185 (29.70) | 49 (27.84) | 0.034 | 0.980 |
| **Hyperlipidemia (n, %)** | 290 (15.56) | 260 (17.78) | 30 (7.46) | ＜0.001 |  | 119 (14.89) | 110 (17.66) | 9 (5.11) | ＜0.001 | 0.663 |
| **Hyperuricemia (n, %)** | 73 (3.92) | 64 (4.38) | 9 (2.24) | 0.050 |  | 29 (3.63) | 26 (4.17) | 3 (1.70) | 0.122 | 0.724 |
| **Hyperhomocysteinemia (n, %)** | 374 (20.06) | 261 (17.85) | 113 (28.11) | ＜0.001 |  | 134 (16.77) | 100 (16.05) | 34 (19.32) | 0.002 | 0.057 |
| **Coronary heart disease (n, %)** | 221 (11.86) | 142 (9.71) | 79 (19.65) | ＜0.001 |  | 100 (12.52) | 69 (11.08) | 31 (17.61) | 0.021 | 0.632 |
| **Atrial fibrillation (n, %)** | 274 (14.70) | 175 (11.97) | 99 (24.63) | ＜0.001 |  | 115 (14.39) | 69 (11.08) | 46 (26.14) | ＜0.001 | 0.837 |
| **Cardiac insufficiency (n, %)** | 189 (10.14) | 96 (6.57) | 93 (23.13) | ＜0.001 |  | 74 (9.26) | 40 (6.42) | 34 (19.32) | ＜0.001 | 0.487 |
| **Renal insufficiency (n, %)** | 113 (6.06) | 68 (4.65) | 45 (11.19) | ＜0.001 |  | 56 (7.01) | 39 (6.26) | 17 (9.66) | 0.019 | 0.359 |
| **Hepatic insufficiency (n, %)** | 141 (7.56) | 94 (6.43) | 47 (11.69) | 0.004 |  | 59 (7.38) | 35 (5.62) | 24 (13.64) | ＜0.001 | 0.872 |
| **FBG [mmol/L, M (P25, P75)]** | 7.13 (5.47, 8.39) | 6.60 (5.33, 7.65) | 7.53 (6.98, 11.38) | ＜0.001 |  | 7.13 (5.53, 8.61) | 6.82 (5.36, 7.82) | 7.85 (7.10, 11.95) | ＜0.001 | 0.246 |
| **FBG (n, %)** |  |  |  |  |  |  |  |  |  |  |
| **4-7** | 904 (48.50) | 803 (54.92) | 101 (25.12) | ＜0.001 |  | 365 (45.68) | 326 (52.33) | 39 (22.16) | ＜0.001 | 0.182 |
| **＜4 or ＞7** | 960 (51.50) | 659 (45.08) | 301 (74.88) |  |  | 434 (54.32) | 297 (47.67) | 137 (77.84) |  |  |
| **CRP [mg/L, M (P25, P75)]** | 4.51 (0.00, 36.11) | 2.35 (0.00, 24.09) | 63.29 (14.22, 123.19) | ＜0.001 |  | 4.56 (0.00, 34.51) | 2.19 (0.00, 19.43) | 61.17 (21.09, 116.07) | ＜0.001 | 0.383 |
| **CRP (n, %)** |  |  |  |  |  |  |  |  |  |  |
| **＜10** | 1061 (56.92) | 968 (66.21) | 93 (23.13) | ＜0.001 |  | 463 (57.95) | 424 (68.06) | 39 (22.16) | ＜0.001 | 0.734 |
| **10-19** | 122 (6.55) | 103 (7.05) | 19 (4.73) |  |  | 49 (6.13) | 45 (7.22) | 4 (2.27) |  |  |
| **20-29** | 180 (9.66) | 153 (10.47) | 27 (6.72) |  |  | 71 (8.89) | 59 (9.47) | 12 (6.82) |  |  |
| **30-39** | 57 (3.06) | 40 (2.74) | 17 (4.23) |  |  | 33 (4.13) | 23 (3.69) | 10 (5.68) |  |  |
| **40-49** | 47 (2.52) | 30 (2.05) | 17 (4.23) |  |  | 18 (2.25) | 7 (1.12) | 11 (6.25) |  |  |
| **≥50** | 397 (21.30) | 168 (11.49) | 229 (56.97) |  |  | 165 (20.65) | 65 (10.43) | 100 (56.82) |  |  |
| **WBC [×10^9^/L, M (P25, P75)]** | 8.09 (6.20, 11.73) | 7.50 (5.90, 10.00) | 12.80 (9.32, 17.30) | ＜0.001 |  | 8.20 (6.10, 11.60) | 7.50 (5.80, 9.40) | 13.40 (10.30, 16.60) | ＜0.001 | 0.288 |
| **WBC (n, %)** |  |  |  |  |  |  |  |  |  |  |
| 4-10 | 1160 (62.23) | 1050 (71.82) | 110 (27.36) | ＜0.001 |  | 503 (62.95) | 465 (74.64) | 38 (21.59) | ＜0.001 | 0.724 |
| ＜4 or ＞10 | 704 (37.77) | 412 (28.18) | 292 (72.64) |  |  | 296 (37.05) | 158 (25.36) | 138 (78.41) |  |  |
| **NE% [%, M (P25, P75)]** | 73.70 (64.10, 86.30) | 68.95 (61.83, 80.70) | 87.70 (81.86, 91.20) | ＜0.001 |  | 73.70 (62.90, 86.50) | 68.44 (60.85, 81.00) | 88.46 (81.90, 91.73) | ＜0.001 | 0.913 |
| **NE% (n, %)** |  |  |  |  |  |  |  |  |  |  |
| ＜80 | 1158 (62.12) | 1074 (73.46) | 84 (20.90) | ＜0.001 |  | 489 (61.20) | 456 (73.19) | 33 (18.75) | ＜0.001 | 0.653 |
| ≥80 | 706 (37.88) | 388 (26.54) | 318 (79.10) |  |  | 310 (38.80) | 167 (26.81) | 143 (81.25) |  |  |
| **Hb [g/L, M (P25, P75)]** | 124.83 (110.00, 135.25) | 125.00 (115.00, 137.00) | 109.00 (89.00, 125.00) | ＜0.001 |  | 123.00 (107.00, 135.00) | 124.83 (113.0, 137.50) | 109.00 (82.75, 124.87) | ＜0.001 | 0.086 |
| **Hb (n, %)** |  |  |  |  |  |  |  |  |  |  |
| ≥120 | 1120 (60.09) | 978 (66.89) | 142 (35.32) | ＜0.001 |  | 461 (57.70) | 397 (63.72) | 64 (36.36) | ＜0.001 | 0.269 |
| 119-90 | 563 (30.20) | 406 (27.77) | 157 (39.05) |  |  | 241 (30.16) | 183 (29.37) | 58 (32.95) |  |  |
| 89-60 | 161 (8.64) | 71 (4.86) | 90 (22.39) |  |  | 88 (11.01) | 39 (6.26) | 49 (27.84) |  |  |
| ＜60 | 20 (1.07) | 7 (0.48) | 13 (3.23) |  |  | 9 (1.13) | 4 (0.64) | 5 (2.84) |  |  |

Note: P***^$^*** means the P-value between two groups in the training cohort. P***^&^*** means the P-value between two groups in the validation cohort. P***^#^*** means P-value for intergroup comparison (AP vs. Non-AP) in the whole cohort (Training + Validation group).
