# Supplementary material for: Innovative nomogram for predictive risk stratification of aspiration pneumonia in post-stroke dysphagia patients
Source: Front Neurol. 2025 Jun 3;16:1556541. doi: 10.3389/fneur.2025.1556541 (PMC12170325; doi:10.3389/fneur.2025.1556541)
Supplement: Supplementary file 6 [file Table_6.docx]

**Supplementary Table 6 Comparison of general clinical data between Non-AP group and AP group PSD patients**

| **Factors** | **Non-AP**  **(n = 388)** | **AP**  **(n = 112)** | **Total**  **(n = 500)** | ***t or x^2^*** | ***P*** |
| --- | --- | --- | --- | --- | --- |
| **Gender (n, %)** |  |  |  | 0.978 | 0.323 |
| **Female** | 153 (39.43) | 50 (44.64) | 203 (40.60) |  |  |
| **Male** | 235 (60.57) | 62 (55.36) | 297 (59.40) |  |  |
| **Age [Years, M(P25, P75)]** | 68 (63, 77) | 75 (55, 82) | 69 (60, 7) | 3.179 | 0.002 |
| **Length of hospital stay [Days, M(P25, P75)]** | 11 (8, 15) | 16 (9, 27) | 12 (8, 17) | 5.856 | ＜0.001 |
| **Way of discharge (n, %)** |  |  |  | 53.752 | ＜0.001 |
| **Improved** | 333 (85.82) | 60 (53.57) | 393 (78.60) |  |  |
| **Death or non recovery** | 55 (14.18) | 52 (46.43) | 107 (21.40) |  |  |
| **Type of stroke (n, %)** |  |  |  | 11.956 | 0.001 |
| **Ischemic stroke** | 302 (77.84) | 69 (61.61) | 371 (74.20) |  |  |
| **hemorrhagic stroke** | 86 (22.16) | 43 (38.39) | 129 (25.80) |  |  |
| **NIHSS score [Points, M (P25, P75)]** | 3 (0, 3) | 3 (3, 6) | 3 (1, 3) | 4.751 | ＜0.001 |
| **Smoking history (n, %)** | 24 (6.19) | 10 (8.93) | 34 (6.80) | 1.032 | 0.310 |
| **Drinking history (n, %)** | 17 (4.38) | 8 (7.14) | 25 (5.00) | 1.395 | 0.238 |
| **BMI [kg/m2, M (P25, P75)]** | 24.0 (23.1, 25.3) | 24 (22.8, 25.7) | 24 (22.9, 25.3) | 0.685 | 0.494 |
| **Vital signs (n, %)** |  |  |  |  |  |
| **Body temperature (℃)** |  |  |  | 2.670 | 0.102 |
| **36-37** | 333（85.82） | 89（79.46） | 422（84.40） |  |  |
| **＜36 or ＞37** | 55（14.18） | 23（20.54） | 78（15.60） |  |  |
| **Pulse (Per min)** |  |  |  | 4.535 | 0.033 |
| **60-100** | 375（96.64） | 103（91.96） | 478（95.60） |  |  |
| **＜60 or ＞100** | 13（3.35） | 9（8.04） | 22（4.40） |  |  |
| **Breathe (Per min)** |  |  |  | 21.521 | ＜0.001 |
| **12-20** | 348（89.69） | 81（72.32） | 429（85.80） |  |  |
| **＜12 or ＞20** | 40（10.31） | 31（27.68） | 71（14.20） |  |  |
| **Blood pressure (mmHg)** |  |  |  | 0.104 | 0.747 |
| **Systolic pressure＜140 and diastolic pressure＜90** | 117（30.15） | 32（28.57） | 149（29.80） |  |  |
| **Systolic pressure≥140 or diastolic pressure≥90** | 271（69.85） | 80（71.43） | 351（70.20） |  |  |
| **Underlying disease (n, %)** |  |  |  |  |  |
| **Encephalatrophy** | 72（18.56） | 11（9.82） | 83（16.60） | 4.790 | 0.028 |
| **Hypertension** | 276（71.13） | 76（67.86） | 352（70.40） | 0.448 | 0.503 |
| **Diabetes** | 129（33.25） | 25（22.32） | 154（30.80） | 4.868 | 0.027 |
| **Hyperlipidemia** | 62（15.98） | 7（6.25） | 69（13.80） | 6.916 | 0.009 |
| **Hyperhomocysteinemia** | 80（20.62） | 34（30.36） | 114（22.80） | 4.683 | 0.030 |
| **Coronary heart disease** | 40（10.31） | 23（20.54） | 63（12.60） | 8.254 | 0.004 |
| **Atrial fibrillation** | 47（12.11） | 26（23.21） | 73（14.60） | 8.590 | 0.003 |
| **Cardiac insufficiency** | 29（7.47） | 19（16.96） | 48（9.60） | 9.019 | 0.003 |
| **Pulmonary underlying diseases** | 24（6.19） | 9（8.04） | 33（6.60） | 0.483 | 0.487 |
| **Hepatic insufficiency** | 23（5.93） | 12（10.71） | 35（7.00） | 3.059 | 0.080 |
| **Renal insufficiency** | 18（4.64） | 15（13.39） | 33（6.60） | 10.804 | 0.001 |
| **Malignant tumors history** | 11（2.84） | 4（3.57） | 15（3.00） | 0.162 | 0.687 |
