# Supplementary material for: Innovative nomogram for predictive risk stratification of aspiration pneumonia in post-stroke dysphagia patients
Source: Front Neurol. 2025 Jun 3;16:1556541. doi: 10.3389/fneur.2025.1556541 (PMC12170325; doi:10.3389/fneur.2025.1556541)
Supplement: Supplementary file 7 [file Table_7.docx]

**Supplementary Table 7 Comparison of important laboratory examination between Non-AP group and AP group PSD patients in the external validation queue**

| **Factors** | **Non-AP**  **（n = 388）** | **AP**  **（n = 112）** | **Total**  **（n = 500）** | ***t or x^2^*** | ***P*** |
| --- | --- | --- | --- | --- | --- |
| **CRP [mg/L, M (P25, P75)]** | 2.48（0.00，29.49） | 91.96（41.37，173.60） | 6.92（0.00，42.90） | 14.460 | ＜0.001 |
| **Routine blood test [M (P25, P75)]** |  |  |  |  |  |
| **WBC (×10^9^/L)** | 7.5（6.00，10.30） | 11.55（9.28，18.20） | 8.09（6.30，11.85） | 11.030 | ＜0.001 |
| **NE% (%)** | 69.75（62.35，81.43） | 87.41（81.73，90.76） | 75.11（65.10，86.40） | 11.060 | ＜0.001 |
| **LY% (%)** | 26.3（19.3，32.3） | 17.8（12.6，25.6） | 24.3（17.0，30.8） | 5.991 | ＜0.001 |
| **NE (×10^9^/L)** | 5.3（3.7，8.2） | 9.8（7.4，15.9） | 5.9（4.0，9.7） | 11.540 | ＜0.001 |
| **LY (×10^9^/L)** | 1.7（1.3，2.0） | 1.42（1.1，2.1） | 1.6（1.2，2.0） | 0.711 | 0.477 |
| **NE/LY** | 3.3（2.1，5.5） | 7.4（5.4，9.8） | 3.9（2.4，6.9） | 7.972 | ＜0.001 |
| **RBC (×10^12^/L)** | 4.2（3.8，4.5） | 3.6（3.0，4.1） | 4.1（3.6，4.5） | 8.463 | ＜0.001 |
| **Hb (g/L)** | 126（115，137） | 111（91，124） | 124（110，135） | 8.606 | ＜0.001 |
| **Hct (L/L)** | 0.37（0.34，0.40） | 0.32（0.27，0.37） | 0.37（0.33，0.40） | 8.148 | ＜0.001 |
| **Plt (×10^9^/L)** | 187（143，230） | 133（99，189） | 178（131，222） | 5.638 | ＜0.001 |
| **WBC/RBC (×10^-3^)** | 1.80（1.39，2.54） | 3.43（2.47，5.49） | 1.97（1.47，3.03） | 12.160 | ＜0.001 |
| **FBG (mmol/L)** | 6.50（5.28，7.46） | 7.30（6.65，11.31） | 6.92（5.45，8.22） | 6.609 | ＜0.001 |
| **GHb (%)** | 5.8（5.8，6.9） | 5.8（5.7，6.5） | 5.8（5.8，6.8） | 2.056 | 0.040 |
| **Blood fat [M (P25, P75)]** |  |  |  |  |  |
| **TC (mmol/L)** | 4.18（3.72，4.94） | 4.18（3.45，4.90） | 4.18（3.69，4.94） | 1.269 | 0.205 |
| **TG (mmol/L)** | 1.37（0.97，1.71） | 1.23（0.78，1.51） | 1.36（0.96，1.68） | 2.635 | 0.009 |
| **LDL (mmol/L)** | 2.85（2.33，3.21） | 2.83（2.12，3.04） | 2.85（2.26，3.18） | 1.446 | 0.149 |
| **HDL (mmol/L)** | 1.08（0.92，1.21） | 1.08（0.95，1.25） | 1.08（0.93，1.23） | 1.002 | 0.317 |
| **Hepatic function [M (P25, P75)]** |  |  |  |  |  |
| **ALT (U/L)** | 27.00（17.75，33.19） | 33.19（21.75，50.00） | 29.00（18.00，34.00） | 3.587 | ＜0.001 |
| **TP (g/L)** | 62.43（60.00，65.00） | 62.43（55.75，63.00） | 62.43（59.00，65.00） | 4.713 | ＜0.001 |
| **Alb (g/L)** | 35.48（34.00，38.00） | 34.00（26.00，35.48） | 35.48（33.00，37.00） | 7.574 | ＜0.001 |
| **PA (mg/L)** | 210.86（182.75，248.25） | 177.00（105.50，210.86） | 210.86（160.00，237.00） | 7.578 | ＜0.001 |
| **Renal function [M (P25, P75)]** |  |  |  |  |  |
| **Scr (umol/L)** | 79.5（65.0，84.4） | 84.4（64.0，102.5） | 80.0（65.0，84.4） | 4.809 | ＜0.001 |
| **GFR [mL/ (min×1.73m^2^)]** | 99.87（62.52，113.30） | 62.52（62.52，113.30） | 92.95（62.52，113.30） | 2.615 | 0.009 |
| **BNP (ng/L)** | 80.71（21.93，145.78） | 172.43（74.01，495.70） | 110.00（27.91，166.73） | 5.711 | ＜0.001 |
| **Electrolyte [M (P25, P75)]** |  |  |  |  |  |
| **K^+^ (mmol/L)** | 3.94（3.79，4.14） | 4.21（3.94，4.60） | 3.94（3.82，4.22） | 7.210 | ＜0.001 |
| **Na^+^ (mmol/L)** | 143.00（141.00，144.00） | 145.00（143.00，153.00） | 143.00（141.00，145.00） | 8.869 | ＜0.001 |
